# Correlative SICM-FCM reveals changes in morphology and kinetics of endocytic pits induced by disease-associated mutations in dynamin

**DOI:** 10.1096/fj.201802635R

**Published:** 2019-04-24

**Authors:** Tayyibah Ali, Joanna Bednarska, Stéphane Vassilopoulos, Martin Tran, Ivan A. Diakonov, Azza Ziyadeh-Isleem, Pascale Guicheney, Julia Gorelik, Yuri E. Korchev, Mary M. Reilly, Marc Bitoun, Andrew Shevchuk

**Affiliations:** * Division of Experimental Medicine, Department of Medicine, Imperial College London, London, United Kingdom;; † Research Center for Myology, Institut de Myologie, UMRS 974, INSERM, Sorbonne Université, Paris, France;; ‡ Department of Cardiac Medicine, National Heart and Lung Institute, Imperial College London, London, United Kingdom;; § UMRS 1166, INSERM, Institute of Cardiometabolism and Nutrition (ICAN), Paris, France;; ¶ UMRS 1166, Sorbonne Universités–Pierre and Marie Curie University (UPMC), Paris, France;; ‖ MRC Centre for Neuromuscular Diseases, UCL Institute of Neurology, Queen Square London, United Kingdom

**Keywords:** clathrin, caveolin, myopathy, Charcot-Marie-Tooth

## Abstract

Dynamin 2 (DNM2) is a GTP-binding protein that controls endocytic vesicle scission and defines a whole class of dynamin-dependent endocytosis, including clathrin-mediated endocytosis by caveoli. It has been suggested that mutations in the *DNM2* gene, associated with 3 inherited diseases, disrupt endocytosis. However, how exactly mutations affect the nanoscale morphology of endocytic machinery has never been studied. In this paper, we used live correlative scanning ion conductance microscopy (SICM) and fluorescence confocal microscopy (FCM) to study how disease-associated mutations affect the morphology and kinetics of clathrin-coated pits (CCPs) by directly following their dynamics of formation, maturation, and internalization in skin fibroblasts from patients with centronuclear myopathy (CNM) and in Cos-7 cells expressing corresponding dynamin mutants. Using SICM-FCM, which we have developed, we show how p.R465W mutation disrupts pit structure, preventing its maturation and internalization, and significantly increases the lifetime of CCPs. Differently, p.R522H slows down the formation of CCPs without affecting their internalization. We also found that CNM mutations in *DNM2* affect the distribution of caveoli and reduce dorsal ruffling in human skin fibroblasts. Collectively, our SICM-FCM findings at single CCP level, backed up by electron microscopy data, argue for the impairment of several forms of endocytosis in *DNM2*-linked CNM.—Ali, T., Bednarska, J., Vassilopoulos, S., Tran, M., Diakonov, I. A., Ziyadeh-Isleem, A., Guicheney, P., Gorelik, J., Korchev, Y. E., Reilly, M. M., Bitoun, M., Shevchuk, A. Correlative SICM-FCM reveals changes in morphology and kinetics of endocytic pits induced by disease-associated mutations in dynamin.

Dynamin 2 (DNM2) is a mechanochemical and regulatory GTPase that defines the whole class of dynamin-dependent endocytosis mechanisms, including clathrin-mediated (CME), caveolin-mediated, and raft-dependent endocytosis (1–3). It also plays a crucial role in actin and membrane remodelling (4). Autosomal-dominant centronuclear myopathy (CNM) (5), dominant Charcot-Marie-Tooth disease (CMT) (6), and autosomal-dominant hereditary spastic paraplegia (HSP) (7) are 3 distinct neuromuscular disorders associated with mutations in *DNM2* gene. Also, mutations in *DNM2* were found to be associated with acute lymphoblastic leukemia (8). *DNM2*-related CNM is a slowly progressive congenital myopathy resulting in generalized muscle weakness with variable severity, ranging from severe neonatal to mild late-onset forms (9–11). CMT is an inherited peripheral neuropathy characterized by muscle weakness and atrophy and loss of touch sensation, which occurs in 1 in 2500 people, making it currently one of the most common incurable inherited neurologic disorders. Mutations in *DNM2* have been identified in the axonal CMT (CMT2) and dominant intermediate forms of CMT (6, 12). HSP is a large group of clinically and genetically heterogeneous disorders resulting in progressive gait disorder because of the dysfunction of long axons in the spinal cord (13).

Currently, the commonly accepted model of dynamin action in CME depicts a highly orchestrated involvement of all of its domains in the formation and constricting dynamin helix around the neck of invaginated clathrin-coated pits (CCPs) to achieve complete pit separation from the cell membrane and form an endocytic vesicle (14). Based on the spatial-temporal correlation of clathrin, dynamin, and membrane cargo fluorescence puncta in live cells, it was suggested that dynamin plays a regulatory role in CME, starting from the very early stages of CCP nucleation (15–18). DNM2 is formed of a GTPase domain that binds and hydrolyses GTP, a middle domain (MD) that is responsible for the assembly of rings and helixes, a pleckstrin-homology (PH) domain that binds phosphoinositides and is responsible for the targeting of dynamin to the plasma membrane, a GTPase effector domain that regulates GTPase activity and self-assembly, and a proline-rich domain mediating protein-protein interactions. To date, 10 heterozygous mutations associated with CMT, 24 with CNM, and 1 in HSP were identified in MD, PH, GTPase effector domain, and proline-rich domain with no common mutations to these 3 disorders.

Based on the early findings that transferrin (Tfn) receptor is internalized predominantly *via* CCPs (19, 20), researchers traditionally used fluorescently labeled or biotinylated Tfn uptake as a measure of CME efficiency. In these assays, the total amount of internalized Tfn molecules is measured in cell lysates or chemically fixed preparations. Several attempts to test whether the disease-associated mutations in *DNM2* affect CME levels using Tfn uptake produced contradictory results. For example, p.R465W mutation in MD has been reported to result in significant reduction in Tfn uptake by Bitoun *et al.*(11) and Koutsopoulos *et al.* (21), but not by Liu *et al.* (22) or Sidiropoulos *et al.*(23), who reported no difference in the uptake levels.

The effect of *DNM2* mutations on endocytosis can also be studied by imaging endocytic pits and vesicles in fixed preparations using various electron microscopy (EM) techniques or in living cells by fluorescence live imaging such as confocal or total internal reflection fluorescence microscopy. Though EM is capable of ultra-high resolution sufficient to depict CCP structure at close to molecular level, it cannot provide important information on the kinetics of endocytic vesicle formation. In contrast, fluorescence imaging techniques have an acquisition rate appropriate to follow CCP dynamics in real time but lack the resolution necessary to resolve CCP structure. Advances in super resolution optical imaging, such as stimulated emission depletion (STED), stochastic optical reconstruction microscopy, structured illumination microscopy, and lattice light sheet microscopy (LLSM), now allow for the detection of fluorescently labeled molecules with a resolution of tens of nanometers (24–27). However, the ability of detecting nothing but fluorescent tags attached to structures of interest with certain affinity is the greatest limitation of fluorescence microscopy. Super resolution fluorescence microscopy has been successfully applied to image endocytosis in live cells (26, 28), although until recently it was difficult to correlate molecular-specific fluorescence data to cell membrane nanostructures. This is because the visualization of the cell membrane topography by live imaging techniques remains challenging. Collectively, the above limitations resulted in remarkable heterogeneity of putative CCPs because fluorescence signals from clathrin contained in genuine CCPs, flat lattices, and endosomes are difficult to distinguish (29, 30). Recently, developments in fluorescence probes for phospholipid components of cell membranes have enabled detailed characterization of vesicle fusion and fission kinetics and helped to elucidate the role of dynamin in fusion pore dynamics in live cells at ∼60 nm spatial and subsecond temporal resolution using STED (31, 32). Also, LLSM was used to follow membrane remodelling and track CCPs and dynamin in live cells at 5.7 s per frame temporal and 150 × 280 nm *xz* resolution (33, 34).

Correlative superresolution fluorescence and metal-replica transmission EM (TEM) has recently been proven suitable for locating fluorescently labeled proteins on the landscape of the cellular plasma membrane at the scale of 20 nm (35). Although researchers have demonstrated clear colocalization between AF647-labeled clathrin and CCP structures, the technique is not capable of studying CCP kinetics. The lack of live and dynamic investigations in cell membrane morphologic changes, resulting from the recruitment of specific molecules, is a limitation of current imaging techniques, which has been previously highlighted (36, 37).

Previously, we have developed an imaging technique that, in contrast to microscopies described above, enables us to follow morphologic changes of endocytic pits through their entire lifecycle in living cells at close to few nanometers resolution (38). This technique is based on the combination of scanning ion conductance microscopy (SICM) invented by Hansma and colleagues (39) and later adapted to be capable of live imaging (40, 41). SICM is a scanning probe microscopy that generates high-resolution topographical images of samples immersed in liquid by raster scanning a glass nanopipette (probe) that follows the surface of a sample very closely without touching. Importantly, SICM imaging does not require any kind of chemical processing of the studied sample and effectively operates in physiologic conditions. This fully noninvasive aspect of imaging makes SICM ideal for living cell studies. The SICM instrument (**Fig. 1*A***) consists of a glass nanopipette mounted on a Z piezo actuator connected to a scan control unit (data not shown) that performs vertical measurements. The same unit also controls raster scanning by moving XY piezo actuator that carries the cell sample in a Petri dish. The ion current that flows between the probing and the reference electrodes located inside the SICM pipette and in the bath, respectively (Fig. 1*B*), is constantly measured by the control electronics. When the pipette vertical position (Fig. 1*C*, top trace) approaches the sample surface to a distance equal to ∼1 radius of pipette opening, the ion current (*I*_pip_) drops (Fig. 1*C*, bottom trace). This drop is used by the control software to stop the approach and record the vertical position of Z piezo actuator as the sample height at a given imaging point. Then, the pipette is withdrawn, and the sample is moved to the next location or pixel. Simultaneous fluorescence confocal imaging is performed by focusing a laser beam at the pipette tip (42, 43). This provides colocalized fluorescence excitation, which is then recorded by a photomultiplier. Confocal autofocusing is achieved by vertical movement of the objective-focusing piezo actuator that is synchronized with the SICM height measurement by Z piezo actuator. This eliminates the need for numerous confocal slicing and subsequent 3-dimensional (3D) reconstruction and allows the fluorescence data to be acquired precisely from the apical membrane in a single scan (red dashed line, Fig. 1*B*). SICM topographical 3D images can be shown either in 2-dimensional view with the height color coded so that the lower areas appear darker and higher areas lighter or as 3D projections. Figure 1*D* shows the example of a 3D topography scan of a cell cytoplasmic membrane where the CCP can be seen as dark invagination (left panel) as well as corresponding fluorescence of clathrin light chain (CLC) a–green fluorescent protein (GFP) (right panel).

**Figure 1:**
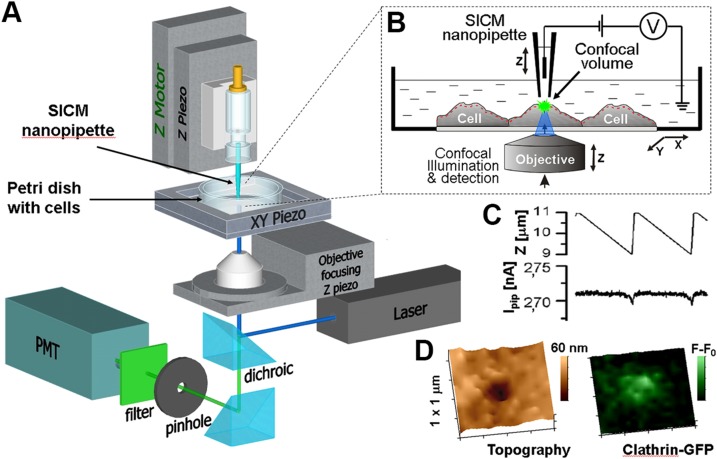
Schematic diagram and principle of operation of SICM-FCM imaging setup. *A*) Diagram showing SICM nanopipette mounted on Z piezo actuator and positioned above living cells grown in a Petri dish. The sample stage is raster scanned in horizontal plane by the XY piezo. The nanopipette is aligned to be coaxial with the laser beam that is fed through the inverted microscope objective to enable simultaneous, correlative topographical and fluorescence confocal imaging. The same objective is used to collect the excited fluorescence, which is then detected by the photomultiplier (PMT). *B*) Principle of SICM operation diagram showing the cross section of the setup arrangement. The laser beam (blue) is focused at the tip of the nanopipette where it (beam) creates confocal volume and excites fluorescence (green). Bias voltage is applied between the measuring electrode inside the nanopipette and the reference electrode in the dish and results in ion current that drops to predefined set-point every time pipette approaches the cell surface. *C*) Experimental measurement showing the trace of the vertical (Z) piezo position during hopping (top trace) and corresponding ion current drop at the lowest point of each approach (bottom). *D*) Example images of CCP seen as an indentation in 3D topography (left) and corresponding CLC-GFP fluorescence image (right).

Integration of SICM with fluorescence confocal microscopy (FCM) together with 2 orders of magnitude improvement in topographical resolution (38) and higher imaging rates made it possible to correlate live observations of nanoscale changes in cell membrane morphology with the localized recruitment of fluorescently labeled molecules of interest. Using correlative SICM-FCM, the recruitment of DNM2-GFP to topographically resolved CCPs has been demonstrated for the first time (44). This technique has also enabled the discovery and characterization of an alternative mechanism of actin-facilitated CCP closure that was more recently confirmed by high-speed atomic force microscopy combined with confocal laser scanning unit (36). Although temporal resolution of SICM remains slower compared with high-speed atomic force microscopy, STED, and LLSM, its topographical (*i.e.*, spatial) resolution of live cell membranes is higher (45).

Analysis of the dynamics of nanoscale morphologic changes taking place in a single trafficking event should be critical to decipher the real influence of pathogenic *DNM2* mutations on CME. With this objective, we report here the use of innovative correlative SICM-FCM to study CME in skin fibroblasts from CNM patients harboring 2 distinct heterozygous *DNM2* mutations and in Cos-7 cells expressing corresponding DNM2-GFP mutants.

## MATERIALS AND METHODS

### SICM instrumentation

All experiments were performed using the SICM setup that is capable of better than 20 nm topographical resolution on live cells as previously reported (45). The instrument was controlled by in-house-written software that was also used for data acquisition and analysis. The SICM imaging head controlled by SICM scanner controller (Ionscope, Cambridge, United Kingdom) was built using P-733.2DD XY Piezo-Nanopositioning Stage (PI, Karlsruhe, Germany) 40 µm travel (*xy* movement of the sample) and P-753.21 piezo actuator (PI) 25 µm travel (*z* movement of pipette). Piezo stages were driven by 200 W peak-power low-voltage PZT amplifier E-505 (PI) in capacitive sensor-controlled closed-loop using Sensor & Position Servo-Control Module E-509 (PI). The XY piezo scanner was incorporated into a heavy stainless-steel platform, which was placed onto an inverted Nikon TE2000-U microscope (Nikon, Tokyo, Japan) table spring preloaded and equipped with differential micrometers (OptoSigma, Santa Ana, CA, USA) for precise positioning. Coarse positioning of the pipette in the *z* axis was provided by a M-112.1DG translation stage with a travel range of 25 mm that was coupled with a crossed roller linear translation stage (OptoSigma) to improve stability. To reduce the vibrations caused by the resonance of the glass pipette, standard ESW-F10P holder (Warner Instruments, Hamden, CT, USA) was replaced with a V-grove mounting plate where the pipette was held by a steel spring ∼7 mm above the taper. Nanopipettes were pulled from borosilicate glass (outer diameter, 1 mm; inner diameter, 0.5 mm; Intracell, Saint Ives, United Kingdom) using a laser-based puller Model P-2000 (Sutter Instruments, Novato, CA, USA). The pipettes displayed resistances of ∼400 MΩ (range 300–500 MΩ.) and had an estimated inner diameter of 30 nm. The pipette inner diameters are estimated from the pipette resistance using a half cone angle of 3°. Pipette current was detected *via* an Axopatch 200B (Molecular Devices, Sunnyvale, CA, USA) using a gain of 1 mV/pA and a low-pass filter setting of 5 kHz. The internal holding voltage source of the Axopatch 200B was used to supply a DC voltage of +200 mV to the pipette. The outputs of the capacitive sensors from all 3 piezo elements were monitored using Axon Digidata 1322A (Molecular Devices) and Clampex 9.2 (Molecular Devices). Correlative fluorescence images were recorded using D-104 Microscope Photometer (Photon Technology International, Birmingham, NJ, USA) through a ×100/1.3 oil immersion objective. The excitation was provided by Protera 488 nm wavelength diode-pumped solid-state laser (Laser 200, Huntingdon, United Kingdom). SICM-FCM imaging was done either in Leibovetz’s L 15 or CO_2_ Independent medium (Thermo Fisher Scientific, Waltham, MA, USA). Combined fluorescence and topographical imaging was done in Phenol-Red–free Leibovetz’s L 15 medium (Thermo Fisher Scientific). The temperature of the medium in the bath was measured with a CL-100 temperature controller (Warner Instruments) as 28 ± 1°C. This temperature that was higher than ambient room temperature was caused by the heat emitted by the piezo scanner, epi-fluorescent attachment, and other surrounding electronic equipment.

### Cell culture and plasmids

Skin fibroblasts from CNM patients harboring the p.R465W (c.1393C > T; skin biopsy at the age of 30 yr) or the p.R522H mutation (c.1565G > A; skin biopsy at the age of 20 yr) and skin fibroblasts from healthy subjects (biopsied at the ages of 26 and 30 for controls 1 and 2) were cultured in DMEM supplemented with 10% fetal calf serum (FCS) in a 5% CO_2_ incubator at 37°C. The p.R465W fibroblast cell line was obtained from a patient of a previously reported large autosomal-dominant CNM family (11), and the p.R522H fibroblast cell line was obtained from a patient of an unpublished autosomal-dominant CNM family. In both patients, mutations were identified by Sanger sequencing.

Monkey Cos-7 cells were routinely maintained at 37°C in 5% CO_2_ using DMEM (Thermo Fisher Scientific) containing 5% (vol/vol) FCS. The plasmid DNA used in the experiments were pCi (Promega, Madison, WI, USA) containing clathrin-GFP (kindly provided by Dr. Lois E. Greene, Laboratory of Cell Biology, National Heart, Lung, and Blood Institute, National Institutes of Health, Bethesda, MD, USA) (46). The open reading frame of the wild-type (WT) DNM2 isoform 1 (NM_001005360) was generated by RT-PCR from lymphocyte mRNA and inserted in the frame with the GFP in pcDNA3.1/NT-GFP-TOPO (Thermo Fisher Scientific). The R465W and R522H plasmids were generated by directed mutagenesis using the Quick Change Site-Directed Mutagenesis Kit (Agilent Technologies, Santa Clara, CA, USA). The constructed plasmids were verified by DNA sequencing.

### Transfection

Cos-7 cells (1 × 10^6^ cells per flask) were plated into a T25 flask and incubated overnight at 37°C in DMEM containing 5% FCS. Cells were washed prior to transfection with PBS, and complexes of Lipofectamine 2000 (Thermo Fisher Scientific) and plasmid DNA at a ratio of 1 μl to 1 μg were added in Opti-MEM (Thermo Fisher Scientific) without FCS to the cells. After 2 h, the medium was replaced with DMEM containing 5% FCS. Cells where then either used for live imaging or fixed for 20 min with 3% formaldehyde containing 5% sucrose.

### Uptake assay

Human skin fibroblasts and Cos-7 cells were grown on borosilicate 13-mm glass coverslips (VWR International, West Chester, PA, USA) to 60–80% confluency in DMEM and 10% FCS. Tfn from human serum AlexaFluor 647 conjugate (Thermo Fisher Scientific) and CTxB FITC (MilliporeSigma, Burlington, MA, USA) were added to the cells at a 20-µg/ml concentration for 5, 10, 20, and 30 min at 37°C. Cells were washed 3 times with PBS, acid-stripped (0.2 M Na_2_HPO_4_, 0.1 M citric acid), and fixed with 4% paraformaldehyde for 10 min. Coverslips were mounted onto glass slides with prolong diamond antifade mountant with DAPI (Thermo Fisher Scientific). Cells were imaged with Zeiss (Oberkochen, Germany) LSM780 inverted confocal microscope. Individual cells were manually outlined, and corrected total cell fluorescence = integrated density − (area of selected cell × mean fluorescence of background readings) was calculated using plugin contributed by Dr. Martin Fitzpatrick (University of Birmingham, Birmingham, United Kingdom) in Fiji (*https://fiji.sc/*) software (47).

### Ultrastructural analysis by TEM

The cells, grown to 80% confluence, were washed twice with PBS and then primary fixed with 2.5% EM-grade glutaraldehyde (Taab Laboratory Equipment, Reading, United Kingdom) in 0.05 M sodium cacodylate buffer (pH 7.2) for 10 min at room temperature. Fixed cells were scraped with a plastic cell scraper and centrifuged at 2000 rpm for 1 min for collection. Supernatant containing glutaraldehyde was removed, and molten agarose (2% wt/vol in distilled water) at 80°C was added to resuspend the cell pellet and left to solidify. The agarose-suspended cell pellets were stored in 2.5% glutaraldehyde in cacodylate buffer at 4°C. The agarose cell pellets were rinsed 3 times in cacodylate buffer for 10 min with gentle rotation. Each agarose pellet was subdivided and postfixed in 1% osmium tetroxide in cacodylate buffer for 1 h. The samples were then washed twice for 5 min in distilled water. Samples were dehydrated by a graded (70–100%) methanol series. The transition to araldite was through propylene oxide. Samples were immersed in 100% propylene oxide twice for 20 min followed by a 50:50 propylene oxide and araldite mixture for 20 min, a 25:75 propylene oxide and araldite mix for 30 min, and 100% araldite for 30 min, and then they were allowed to infiltrate overnight. Samples were then embedded in araldite in molds and polymerization achieved in an embedding oven at 60°C over 72 h. Araldite blocks were trimmed to reveal the embedded sample and 1-µm semithin toluidine blue survey sections cut by microtome (Reichert Ultracut E; Leica Microsystems, Buffalo Grove, IL, USA) for light microscopy. Areas of interest were selected and shaped for ultramicrotomy, and ultrathin (80-nm) sections were cut. The sections were xylene stretched and picked up on 200 mesh thin bar copper grids for TEM. The sections on grids were contrast stained with 1.5% uranyl acetate for 10 min at room temperature and washed 4 times with methanol, followed by staining with lead citrate at room temperature. Observation was by TEM (Hitachi H7000; Hitachi High-Technologies, Tokyo, Japan) operated at 75 kV at the Royal Brompton Hospital.

### TEM on metal replica from unroofed cells

Adherent plasma membranes from human fibroblasts plated on glass coverslips were disrupted by sonication as previously described (48). Glutaraldehyde- and paraformaldehyde-fixed cells were further sequentially treated with OsO_4_, tannic acid, and uranyl acetate prior to dehydration and Hexamethyldisilazane drying (MilliporeSigma). Dried samples were then rotary-shadowed with platinum and carbon with a high vacuum sputter coater (Leica Microsystems). Platinum replicas were floated off the glass by angled immersion into hydrofluoric acid, washed several times by floatation on distilled water, and picked up on formvar- and carbon-coated EM grids. The grids were mounted in a eucentric side-entry goniometer stage of a transmission electron microscope operated at 80 kV (model CM120; Philips, Andover, MA, USA), and images were recorded with a Morada digital camera (Olympus, Tokyo, Japan). Images were processed in Adobe Photoshop (Adobe, San Jose, CA, USA) to adjust brightness and contrast and presented in inverted contrast.

### Western blot

Cell pellets were homogenized in lysis buffer containing 50 mM of Tris-HCl pH 7.5, 150 mM NaCl, 1 mM EDTA, and NP40 1% supplemented with protease inhibitor cocktail 1% (MilliporeSigma). After centrifugation (14,000 *g*, 4°C, 15 min), protein concentration in the supernatant was determined with the BCA Protein Assay Kit (Thermo Fisher Scientific). Twenty micrograms of proteins were mixed with loading buffer (50 mM Tris-HCl, SDS 2%, glycerol 10%, β-mercaptoethanol 1%, and bromophenol blue) and denaturated at 90°C for 5 min. Protein samples were separated on SDS–PAGE 10% and transferred onto PVDF membranes (0.45-µm pore size; Thermo Fisher Scientific) overnight at 100 mA at 4°C. Membranes were blocked for 1 h at room temperature in PBS containing nonfat dry milk 5% and Tween 20 0.1% and then exposed to rabbit polyclonal anti-clathrin heavy chain antibody (Abcam, Cambridge, MA, USA), rabbit polyclonal anti-caveolin 1 (Santa Cruz Biotechnology, Dallas, TX, USA), or rabbit polyclonal anti–glyceraldehyde-3-phosphate dehydrogenase antibody (Santa Cruz Biotechnology) in PBS–Tween 20 0.1% and milk 1% overnight at 4°C. Membranes were rinsed in PBS–Tween 20 0.1% and incubated 1 h with horseradish peroxidase–conjugated secondary antibody (anti-rabbit from Jackson ImmunoResearch, West Grove, PA, USA) in PBS–Tween 20 0.1%. Chemiluminescence was detected using ECL Detection Kit (Merck, Darmstadt, Germany) in a G-Box Imaging System (Ozyme, Paris, France), and signal quantification was performed using ImageJ software (National Institutes of Health, Bethesda, MD, USA).

### Statistics

CCPs lifetimes (LTs) and cell membrane surface area were compared by independent Student’s *t* test. CCP densities, fluorescently labeled Tfn and cholera enterotoxin subunit B (CTxB) uptake levels, and Western blots were compared by Mann-Whitney *U* test.

## RESULTS

In order to investigate how p.R465W (located in MD) and p.R522H (located in PH domain) mutations affect CCP formation, maturation, and closure kinetics, we used the 2 following distinct models. We first confirmed the recruitment of mutant dynamins to the sites of endocytic pit formation and its effect on pit formation, maturation, and closure kinetics in Cos-7 cells transiently transfected with fluorescently labeled DNM2 constructs (DNM2-WT-GFP, DNM2-R465W-GFP, and DNM2-R522H-GFP). Because both CME and endocytosis *via* caveoli are dynamin dependent, DNM2-GFP fluorescence could correlate with topographically detected indentations corresponding to both types of endocytic pits (*i.e.*, CCPs and caveoli). Based on our previously published SICM measurements, average CCP and caveoli opening diameters equal 118 and 70 nm, respectively (42, 44). We have also demonstrated that 88.7% of endocytic pits in Cos-7 cells are clathrin coated, and only 9.35% are caveoli, with the remaining 2% accounting for topographically detected unknown pits that did not have corresponding Clc-GFP or Cav1-GFP fluorescence (42). Therefore, we conclude that if DNM2-GFP fluorescence colocalizes with topographically detected pit that is larger than 100 nm in diameter, it correlates with CCP. Because transient transfection may result in high DNM2 expression levels that might not reflect physiologically relevant effects and could be cytotoxic (23), we also studied the impact of *DNM2* mutations on CME in CNM patient–derived skin fibroblasts that have endogenous levels of mutant and WT DNM2. In order to unambiguously identify CCPs in human skin fibroblasts, cells were transfected with CLC-GFP (46). Based on the previous publications, we assume that because CLC-a itself cannot induce the formation of new CCPs, for which clathrin heavy chain is required, CLC-GFP only labels endogenous CCPs. It would have been impossible to follow the recruitment of mutant DNM2 to the sites of endocytic vesicle formation in human skin fibroblasts transfected with DNM2-GFP because it would interfere with the endogenous mutant DNM2. In both cell types, we measured LT, diameters, and depth of topographically detected pits.

### Effect of p.R465W and p.R522H dynamin mutations on CCP formation and scission in Cos-7 cells

**Figure 2:**
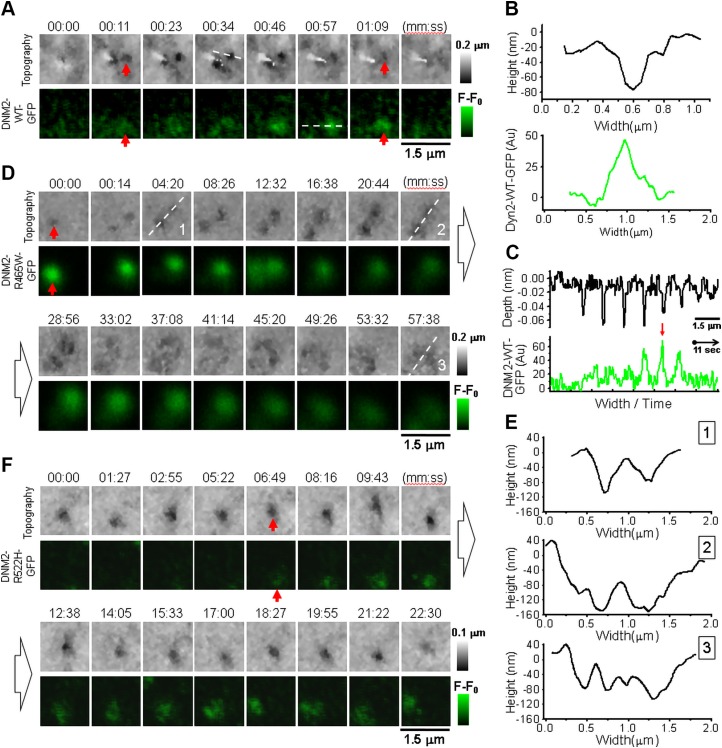
Correlative SICM-FCM time-lapse imaging of CME in Cos-7 cells transfected with DNM2-GFP. *A*) Sequence of topographical (top row) and fluorescence images (bottom row) showing CCP (red arrow) nucleation, maturation, and closure in cells transfected with DNM2-WT-GFP. *B*) Individual cross section profiles of single CCP showing topographically resolved pit width and depth (top trace) and corresponding DNM2-WT-GFP fluorescence. *C*) Series of cross section profiles at locations shown with white dashed lines in sequence *A* concatenated in 1 continuous record, demonstrating highest dynamin recruitment during CCP closure (red arrow). *D*) Sequence of topographical and fluorescence images showing CCP nucleation, widening, and disintegration in cells transfected with mutant DNM2-R465W-GFP. *E*) Topographical cross section profiles of CCP at consecutive stages of disintegration corresponding to white dashed lines in sequence *D* illustrating pit widening. *F*) Sequence of topographical and fluorescence images showing CCP nucleation, widening, and disintegration in cell transfected with mutant DNM2-R522H-GFP.

Correlative SICM-FCM time-lapse images of CCPs in Cos-7 cells transiently transfected with DNM2-WT-GFP (**Fig. 2*A***) revealed similar pit behavior to those in Cos-7 cells transfected with CLC-GFP (44). Topographical SICM images are presented in 2-dimensional view with height color coded so that the lower areas are darker and the higher areas are lighter (Fig. 2*A*, top row). Therefore, CCP is seen as the appearance and disappearance of a dark spot (red arrows) accompanied by corresponding DNM2-WT-GFP fluorescence (Fig. 2*A*, bottom row, and Supplemental Movie S1). The entire CCP life cycle, from nucleation through maturation to closure, had the overall LT of 85.1 ± 25.2 s (*n* = 14, 3 different cells, 2 independent experiments). The top panel in Fig. 2*B* shows the cross section profile of the indentation in the cell membrane corresponding to mature CCP in frame 4 (Fig. 2*A*, top row, white dashed line). The mean opening diameter of mature CCPs measured by SICM as full width at half maximum (in practice, half minimum was used because it corresponds to the depth of pit) was 146 ± 11 nm (*n* = 4). Figure 2*B* (bottom panel) shows DNM2-WT-GFP fluorescence profile at maximum intensity. In order to visualize how CCP formation and scission process develops in time, we plotted the sequence of cross section profiles of CCP topography (top) and corresponding DNM2-WT-GFP fluorescence (bottom) assembled in continuous traces (Fig. 2*C*). The traces show clearly that the topographically resolved formation of CCP was followed by the recruitment of DNM2-WT-GFP fluorescence that reached its maximum at the moment of the pit closure and disappeared abruptly right after (Fig. 2*C*, red arrow). Such kinetics of DNM2 recruitment is in agreement with previously published observations (49).

In Cos-7 cells transiently transfected with DNM2-R465W-GFP, pits successfully went through nucleation to maturation phase, at which they slowed down and started to widen and flatten until finally dissolved (Fig. 2*D* and Supplemental Movie S2). The appearance of DNM2-R465W-GFP fluorescence correlated with the topographically detected pit confirms the recruitment of the mutant dynamin to CCPs at the stage of maturation (red arrows). Because of the recruitment of mutant dynamin to CCPs, the LT of CCPs increased significantly compared with control (1931.6 ± 1219.5 s, *n* = 6, 3 cells, 2 experiments, *P* < 0.005). Also, the recruitment kinetics of mutant dynamin was different from WT, showing high-intensity fluorescence from the very early moments of CCP nucleation. Graphs in Fig. 2*E* show selected, consecutive cross section profiles of 2 adjacent CCPs corresponding to white dashed lines in Fig. 2*D*. The diameter of both pits increased gradually until pits fused into one ∼2-μm–wide, lightly curved structure. By that moment, DNM2-R465W-GFP fluorescence intensity has stopped. Such behavior resembles rather abortive (*i.e.*, non-productive) endocytic events, with pits gradually turning into flat clathrin lattices (FCLs). FCLs were recently described as long-lived structures, also with sustained association with dynamin (50). Similar to p.R465W, the p.R522H mutant dynamin was targeted to the sites of CCP formation, although at a later stage (Fig. 2*F*, red arrows, and Supplemental Movie S3). The LT of CCPs in Cos-7 cells transfected with DNM2-R522H-GFP was also significantly longer compared with control (LT = 928.3 ± 654.4 s, *n* = 7, 4 cells, 4 experiments, *P* < 0.005). Although not to the same extent as in p.R465W, CCPs in cells expressing p.R522H mutant also widen and flatten prior to disappearance. In our imaging experiments with Cos-7 cells expressing mutant dynamins, we did not observe CCPs with WT-like LTs and formation and closing kinetics (R465W: *n* = 95, 7 cells; R522H: *n* = 116, 8 cells).

### Dynamics of CCP formation and scission in skin fibroblasts from CNM subjects with p.R465W and p.R522H dynamin mutations

**Figure 3:**
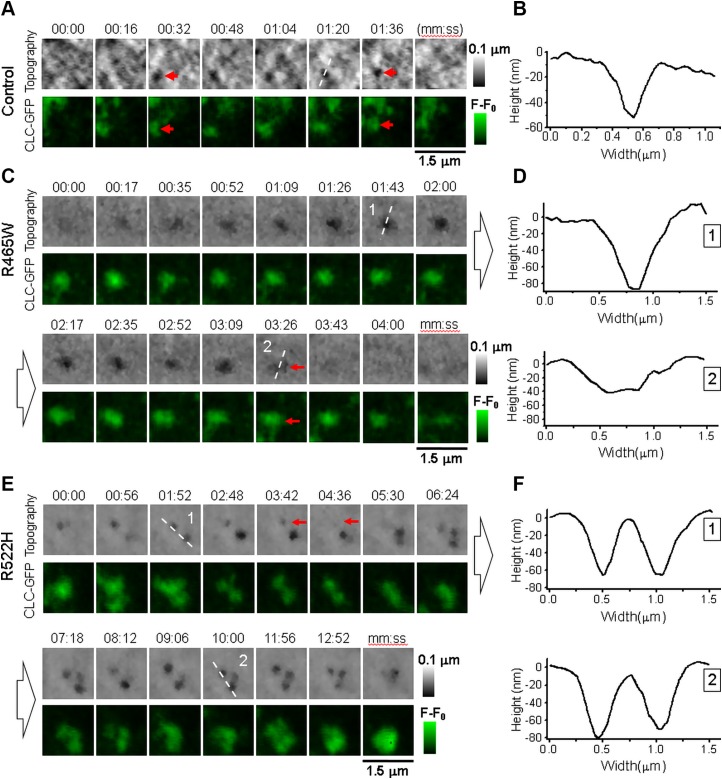
Correlative SICM-FCM time-lapse imaging of CME in human skin fibroblasts transfected with CLC-GFP. *A*) Sequence of topographical (top row) and fluorescence images (bottom row) showing CCP (red arrow) nucleation, maturation, and closure in cells from healthy individuals transfected with CLC-GFP. *B*) Individual cross section profile of CCP corresponding to white dashed line in sequence *A*. *C*) Sequence of topographical and fluorescence images showing CCP nucleation, widening, and disintegration in cells from patients with p.R465W mutation. *D*) Topographical cross section profiles of CCP at consecutive stages of disintegration corresponding to white dashed lines in sequence *C* illustrating pit widening. *E*) Sequence of topographical and fluorescence images showing CCP cluster in cell from patient with p.R522H mutation. *F*) Topographical cross section profiles of CCP corresponding to white dashed lines in sequence *E* illustrating 2 static pits.

We next studied CME in human skin fibroblasts because they express endogenous levels of WT (control) or mutant DNM2. The example of typical CCP nucleation, maturation, and closure in human skin fibroblast from a healthy subject transiently transfected with CLC-GFP is shown in **Fig. 3*A*** (red arrows) and Supplemental Movie S4. Unlike dynamin fluorescence spots that appeared during CCP maturation and peaked at the moment of CCP closure in Cos-7 cells (Fig. 2*A*), CLC-GFP appeared ∼16 s before the topographically detectable invagination corresponding CCP and accompanied the pit throughout its LT in control fibroblasts. The abrupt disappearance of topographically detected indentation that indicates pit closure was followed by the disappearance of associated CLC-GFP fluorescence. The CCP LT was 132.7 ± 85.6 s (*n* = 43, 8 cells, 7 experiments). Figure 3*B* shows a cross section profile corresponding to the white dashed line in Fig. 3*A*, giving pit diameter of 175 nm measured as full width at half maximum. Analysis of CCPs in human skin fibroblasts from patients with p.R465W mutation revealed larger, 380 nm diameter, endocytic structures with significantly longer LTs compared with control fibroblasts (LT = 316.8 ± 220.1 s, *n* = 19, 6 cells, 6 experiments, *P* < 0.005). A typical example of topographically resolved CCP and corresponding CLC-GFP fluorescence in fibroblast with p.R465W mutation is presented in Fig. 3*C* and Supplemental Movie S5. Unlike in cells from healthy individuals, in which the topographically detected pit disappeared abruptly (Fig. 3*A*), in p.R465W cells, the pit gradually widened and flattened (Fig. 3*C*, red arrows). Cross section profiles corresponding to white dashed lines reveal by how much the pit diameter increased and depth reduced during pit degradation (Fig. 3*D*). Such pit behavior is similar to that observed in Cos-7 cells transfected with DNM2-R465W-GFP (Fig. 2*D, E*). In skin fibroblasts from a CNM patient with p.R522H mutation, pits often formed tightly packed clusters (Fig. 3*E* and Supplemental Movie S6). In our experiments, together with 16 individually formed pits, we observed 9 clusters formed by 2–4 CCPs where pits nucleate, mature, and close independently in close vicinity. Although the average CCP LT was significantly longer compared with control (LT = 273.4 ± 192.8 s, *n* = 55, 4 cells, 2 experiments, *P* < 0.005), the abrupt disappearance of individual CCPs and pits in clusters (Fig. 3*E*, red arrow) suggests successful scission events. Figure 3*F* shows the cross section profiles corresponding to white dashed lines in Fig. 3*E*, where 2 static CCPs with longer-than-control LTs can be seen. In our imaging experiments with human skin fibroblasts expressing mutant dynamins, we did not observe CCPs with WT-like LTs and formation and closing kinetics (p.R465W: *n* = 67, 10 cells; p.R522H: *n* = 33, 11 cells).

### Characterization of endocytic structures in cytoplasmic membranes of human skin fibroblasts with p.R465W and p.R522H dynamin mutations by EM

**Figure 4:**
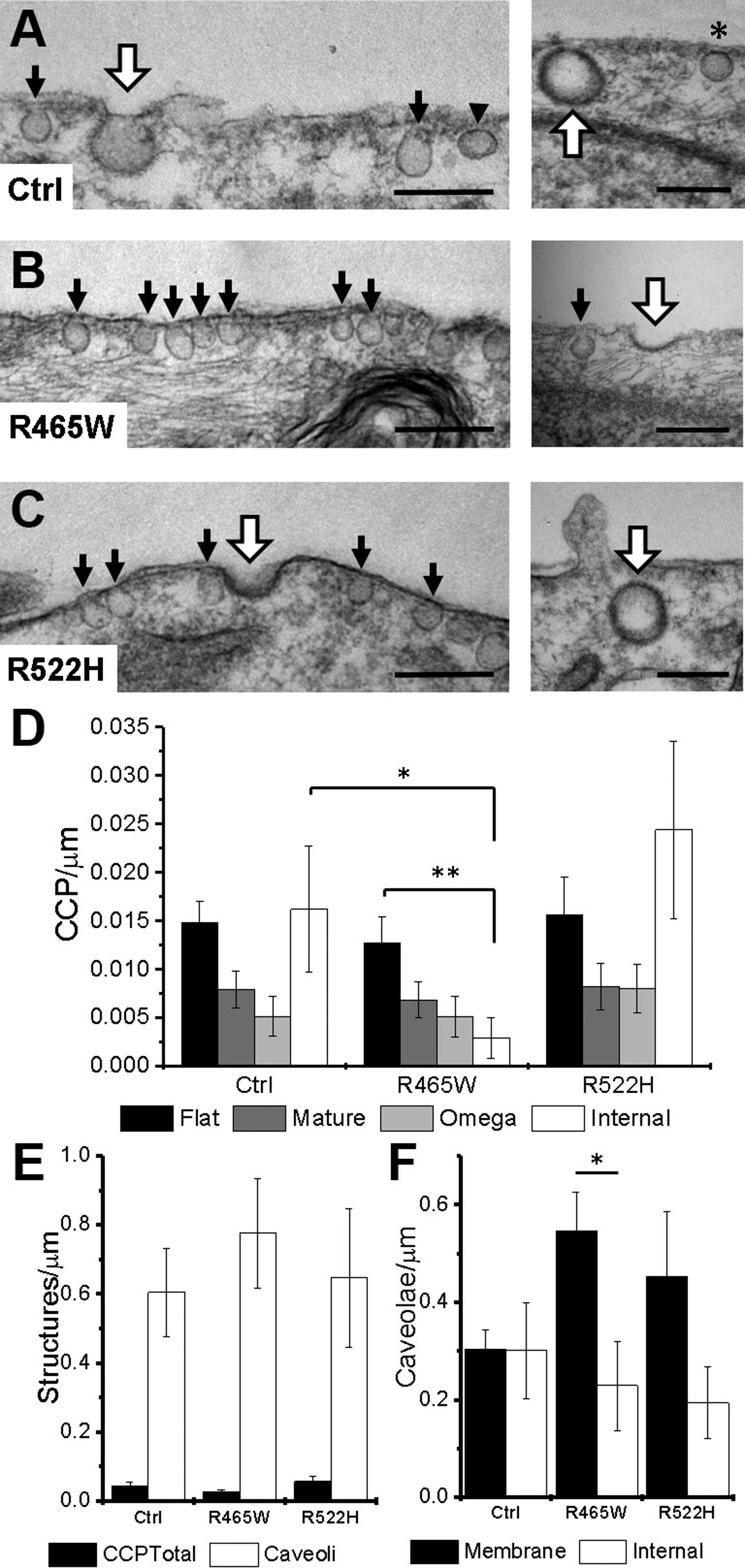
TEM analysis of CCPs and caveoli in human skin fibroblasts. *A–C*) Control (Ctrl; healthy individual) (*A*); cells with p.R465W mutation (*B*); cells with p.R522H mutation (*C*). White bold arrows point at CCPs, black arrows point at caveoli, and arrowhead points at internalized caveoli. Asterisk marks caveolar stomatal diaphragms. Scale bars, 200 nm. *D*) Densities of CCPs at different stages of life cycle. *E*) Densities of total CCPs and caveoli. *F*) Densities of membrane-bound and internalized caveoli. The density is expressed as pits per micrometer of membrane length. *P < 0.05, **P < 0.005.

In order to further characterize endocytic structures in cells from healthy individuals and subjects with p.R465W and p.R522H mutations, and also to confirm our correlative SICM-FCM observations, we used TEM imaging as a well-established, high-resolution imaging technique. High-resolution TEM images of human skin fibroblasts were surveyed along the cytoplasmic membrane for endocytic structures among which CCPs and caveoli were identified (**Fig. 4*A–C***). CCPs at various stages of formation and internalization could be recognized by the characteristic clathrin coat that, in thin sections, appears darker and thicker than the cytoplasmic membrane (bold white arrows). Numerous caveoli could also be seen as round or flask-shaped invaginations docked to the cell membrane that are nearly 2-times smaller than CCPs in diameter (black arrows). Identified CCPs were binned according to the stage of their life cycle as follows: nucleating (*i.e.*, flat or lightly curved), mature or U-shaped (*i.e.*, invaginated to the depth of more than 1 radius of opening), Ω-shaped (*i.e.*, those with constricted opening), and fully internalized (*i.e.*, detached from the membrane). The distribution of densities of CCPs at different stages of life cycle is presented in Fig. 4*D*. Within the control group of cells, the number of nucleating CCPs and internalized clathrin-coated vesicles was insignificantly higher than mature and Ω-shaped CCPs (299 images, total 130 pits, total membrane length analyzed 3787 μm). Such distribution indicates that CCP closure and scission happens quicker than maturation and transportation deeper inside the cell. In contrast, in p.R465W fibroblasts, the number of internalized vesicles was significantly lower than the number of flat CCPs (227 images, total 90 pits, total membrane length analyzed 3409 μm, *P* < 0.05) and the number of internalized vesicles in control fibroblasts (*P* < 0.005). This correlates well with substantially prolonged CCP formation time and eventual pit flattening and disassembly in p.R465W fibroblasts observed by SICM-FCM. In contrast to cells with p.R465W mutation, the distribution of CCPs in p.R522H fibroblasts resembled the one in control cells with an insignificantly higher number of internalized clathrin-coated vesicles (319 images, total 162 pits, total membrane length analyzed 4271 μm). This indicates that despite significantly longer LTs, pits eventually internalize in p.R522H cells as observed by SICM-FCM. The analysis of TEM images was complicated by the fact that the density of CCPs in human skin fibroblasts is low. For comparison, the total numbers of CCPs in control Cos-7 cells is more than 10-times higher. The densities of morphologically identifiable caveoli in human skin fibroblasts were found at least 1 order of magnitude higher than the densities of CCPs at all stages put together (*i.e.*, nucleating through to internalized) in control and both mutant fibroblasts (Fig. 4*E*). The total numbers of identified caveoli in control, p.R465W, and p.R522H fibroblasts were 1906, 1607, and 1626, respectively. The resolution of TEM micrographs was insufficient to identify lightly curved caveoli and distinguish constricted from fully internalized ones; therefore, we could not undertake analysis similar to that of CCPs in full. Hence, we compared the densities of membrane-bound and putatively internalized caveoli (Fig. 4*A*, black arrow and black arrowhead, respectively). In our analysis, only the intracellular vesicles in the vicinity of a half of a micrometer from the morphologically identifiable membrane-bound caveoli were considered internalized caveoli. We found that the total numbers of caveoli in all 3 skin fibroblast lines were roughly similar. Though in control cells, the density of internalized caveoli was the same as membrane-bound ones (*i.e.*, 0.3 ± 0.38 *vs.* 0.3 ± 0.15 caveoli/μm, respectively) (Fig. 4*F*), in p.R465W fibroblasts, the density of internalized caveoli was significantly lower than the membrane-bound ones (0.23 ± 0.3 *vs.* 0.55 ± 0.26 caveoli/μm, *P* < 0.05), and in p.R522H cells, it was also lower, albeit insignificantly (0.19 ± 0.26 *vs.* 0.45 ± 0.5 caveoli/μm, *P* > 0.05).

**Figure 5:**
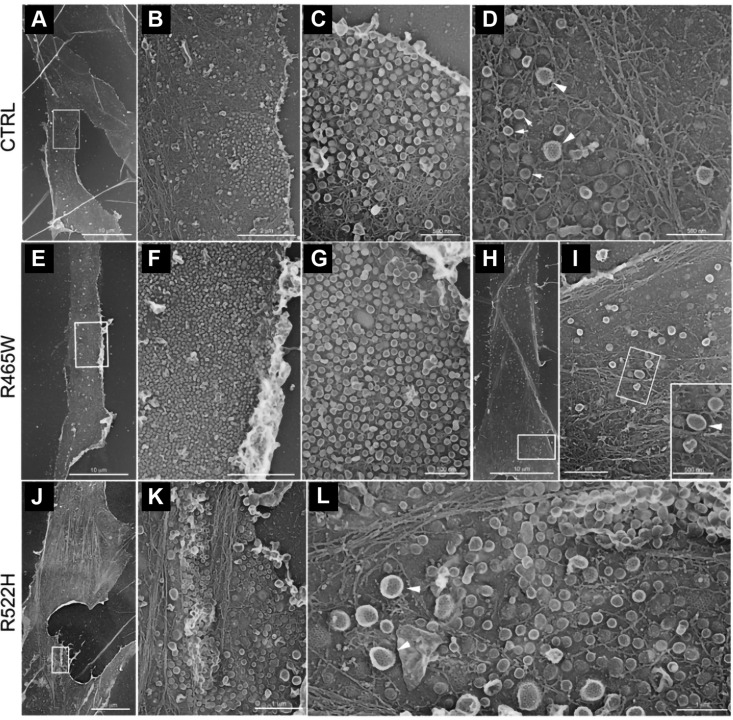
TEM views of the cytoplasmic surface of the plasma membrane from unroofed human skin fibroblasts. Representative images from a healthy individual (*A*–*D*), subject with p.R465W mutation (*E*–*I*), and subject with p.R522H mutation (*J*–*L*) are shown. *A*) Survey view of the cytoplasmic surface of the plasma membrane from unroofed primary human fibroblasts of a healthy individual. *B*) Higher magnification view of caveolae-rich region corresponding to the boxed region in *A*. *C*) Higher magnification view of the caveolae-rich region in *B*. *D*) Higher magnification views of CCPs and caveolae from the cell presented in *C*. *E*) Survey view of the cytoplasmic surface of the plasma membrane from unroofed primary human fibroblasts of a subject with p.R465W mutation. *F*) Higher magnification view corresponding to the boxed region in *E*. *G*) Higher magnification view of the caveolae-rich region in *F*. *H*) Survey view of the cytoplasmic surface of the plasma membrane from unroofed primary human fibroblasts of a subject with R465W mutation. *I*) Higher magnification view of CCPs corresponding to the boxed region in *H*. *J*) Survey view of the cytoplasmic surface of the plasma membrane from unroofed primary human fibroblasts of a subject with p.R522H mutation. *K*) Higher magnification view of caveolae-rich region corresponding to the boxed region in *J*. *L*) Higher magnification views of caveolae and CCPs from the cell pictured in *J*. Arrowheads point at CCPs, and small arrows in *D* point at caveolae. Scale bars, 1 µm (*I*, *K*, *L*); 2 µm (*B*, *F*); 10 µm (*A*, *E*, *H*, *J*); 500 µm [*C*, *D*, *G*, *I* (inset)].

In order to gain further information about the distribution and morphology of CCPs and caveoli at the plasma membrane, EM on metal replica from unroofed cells was performed in healthy control and patient-derived cells. **Figure 5*A–D*** illustrates representative images of the cytoplasmic face of the plasma membrane from control cells. CCPs (white arrowheads) and caveoli (white arrows) were clearly identified by their size and their respective coating. In these control cells, caveoli appeared accumulated in some restricted regions (Fig. 5*B, C*). In p.R465W cells (Fig. 5*E–I*) and p.R522H cells (Fig. 5*J–L*), morphology of CCP and caveoli were similar to the control cells, but regions of caveoli accumulation are larger in p.R465W cells (Fig. 5*F, G*).

### Simultaneous uptake of fluorescently labeled Tfn and cholera toxin b in human skin fibroblasts with p.R465W and p.R522H dynamin mutations

**Figure 6:**
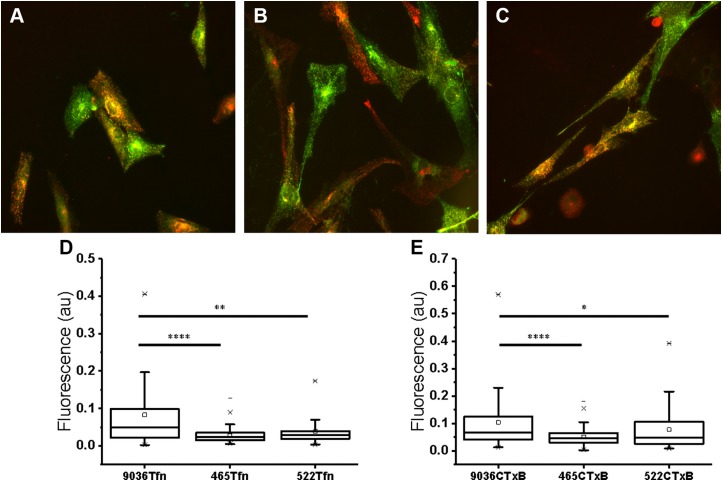
Simultaneous uptake of fluorescently labeled Tfn AlexaFluor 647 (red) and CTxB FITC (green) in human skin fibroblasts. Epi-fluorescence images of fibroblasts. *A*) Control (healthy individual). *B*) Cells with p.R465W mutation. *C*) Cells with p.R522H mutation. Image size 333 × 333 μm (*A*–*C*). *D*, *E*) Comparison of uptake levels of Tfn (*D*) and CTxB (*E*) measured in individual cells; *n* = 79 (control), *n* = 100 (p.R465W), *n* = 69 (p.R522H). *P < 0.05, **P < 0.005, ****P < 0.0001.

In order to confirm whether the mutations in dynamin affect the efficiency of CME and endocytosis by caveoli, we measured simultaneous uptake of AlexaFluor 647-conjugated Tfn from human serum and FITC-conjugated CTxB. This was done assuming that although endocytosis-specific markers such as Tfn receptor, Simian virus, and cholera toxin could be internalized by other independent pathways (51, 52), some preference toward specific pathways exist. Cells were incubated with Tfn and CTxB mix for 5, 10, 20, and 30 min, treated with citric acid to remove surface-bound Tfn and CTxB, fixed, and imaged by Epi-Fluorescence microscopy. Individual cells were manually outlined, and the average fluorescence intensity normalized to a cell surface area was calculated (for details, see Materials and Methods). All cell types were found inhomogeneous in their preference toward Tfn and CTxB uptake (**Fig. 6*A–D***). At 5 and 10 min of incubation, cells didn’t show significant difference in uptake. At 20 and 30 min, the uptake of Tfn and CTxB was found to be significantly lower in human skin fibroblasts with mutations in dynamin (Fig. 6*D, E* correspondingly).

### Clathrin and caveolin expression levels in human skin fibroblasts with p.R465W and p.R522H dynamin mutations

It has previously been demonstrated that DNM2 expression is unchanged in individuals with CNM (5). In order to test whether the difference in the total numbers of observed CCPs and caveoli as well as the amounts of fluorescently labeled Tfn and CTxB uptake is not the result of different clathrin and caveolin expression levels, we performed quantitative Western blotting in human skin fibroblasts (Supplemental Fig. S1). We found that clathrin heavy chain was expressed at significantly higher levels in p.R522H cells compared with controls and p.R465W, and caveolin 1 was significantly higher in both mutants. The results correlate with the number of endocytic structures observed and the amount of fluorescently labeled cargo uptake.

### Morphologic analysis membrane ruffles in apical membranes of human skin fibroblasts induced by p.R465W and p.R522H mutations in DNM2

**Figure 7:**
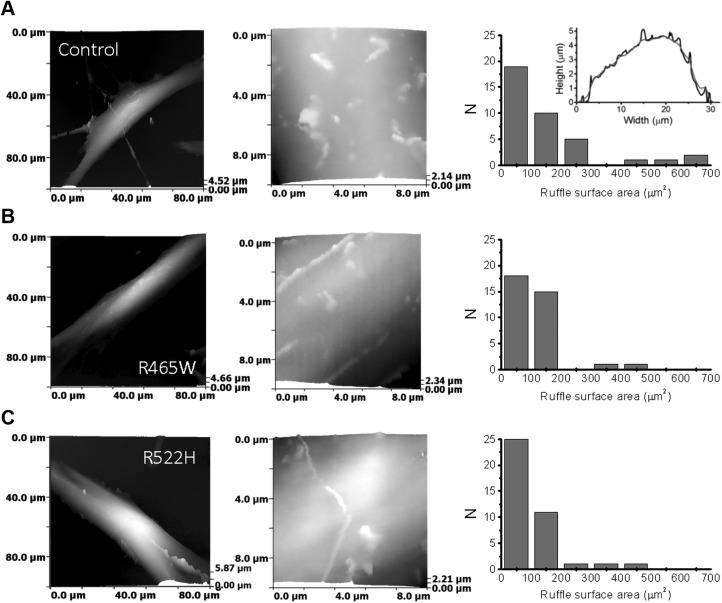
Characterization of human skin fibroblast morphology using SICM topographical imaging. Large-scale SICM 3D topographical images (left column) showing human skin fibroblasts. *A*) Control (healthy individual). *B*) Cells with p.R465W. *C*) Cells with p.R522H mutations. Middle column shows higher-resolution images of corresponding cells, revealing membrane morphology with characteristic membrane ruffling. Right column shows distribution of the membrane surface area stored in membrane ruffles and microvilli calculated as a difference between the cell total surface area and surface area calculated for low-pass filtered cell surface. Inset shows unprocessed (black trace) and low-pass filtered (red trace) cross section profiles of SICM topographical image.

In our previous publication, we have shown that DNM2-GFP colocalizes with roots of highly dynamic microvilli-like protrusions on the cell membrane (44). With this in mind, we tested whether p.R465W and p.R522H mutations induce morphologic changes at a whole-cell and submicrometer level by performing topographical SICM imaging of living human skin fibroblasts. SICM images of control skin fibroblasts (**Fig. 7*A***) and cells with p.R465W and p.R522H (Fig. 7*B, C*) mutations showed similar looking elongated cells with no obvious morphologic differences. Higher-resolution images (Fig. 7, middle column) revealed surface projections resembling dorsal ruffles and microvilli. Because SICM image is a true 3D map of the apical cell surface composed of a limited number of points, in which each point represents cell height at given at *x* and *y* coordinate, the cell surface area can be calculated as a sum of areas of triangles formed by 3 adjacent SICM scan points (53). In order to calculate the surface area accumulated in membrane ruffles and microvilli, we first calculated the surface area of the entire cell in raw SICM images, from which we then subtracted the surface area of the same images subjected to low-pass filtration that removed membrane projections. For illustration, cross section profiles of unprocessed (black) and low-pass filtered (red) control cell is presented in Fig. 7*A* (right column, inset). The distributions of the cell membrane ruffles surface area (Fig. 7, right column) revealed that the control population had a higher number of cells rich in microvilli and ruffles compared with mutants. The median surface area accumulated in membrane ruffles and microvilli was 103.65 μm^2^ for control (*n* = 38), insignificantly lower for p.R465W (72.36 μm^2^, *n* = 35, Mann-Whitney *U* test, *P* = 0.083, 2-tailed), and significantly lower for p.R522H cells (72.17μm^2^, *n* = 39, Mann-Whitney *U* test, *P* = 0.012, 2-tailed). Hence, we conclude that p.R522H mutation may result in the reduction of membrane ruffles density and therefore reduction of nonselective forms of uptake.

## DISCUSSION

The precise mechanism for CME inhibition mediated by CNM and CMT-related DNM2 mutants is still unresolved. Various research techniques used to study this phenomenon produced different types of data that are difficult to link into a coherent model. Using biochemical approaches, it has been demonstrated that p.R465W mutation results in the formation of abnormally stable DNM2 polymers, suggesting that the misbalance in DNM2 assembly-disassembly ratio may disrupt the vesicle release (54). It has also been suggested that it is the impaired GTPase activity that has the inhibitory effect on CME. However, p.R465W and p.S619L (PH domain) mutants that have increased basal and stimulated GTPase activity either result in reduction or have no effect on the fluorescently labeled cargo uptake putatively specific to CME (54–56).

Using the SICM-FCM that we developed, we found that although in p.R465W mutation, CCPs nucleate as normal, they do not proceed to scission but slowly widen and flatten until they finally disintegrate. This is in contrast to WT DNM2, in which we observed rapid formation and abrupt disappearance of the topographically resolved CCPs accompanied by transient burst and disappearance of DNM2-GFP fluorescence. The recruitment of mutant dynamin to the site of CCP maturation was confirmed by the presence of spatially correlated DNM2-R465W-GFP fluorescence. When expressed in Cos-7 cells, p.R465W mutation results in more than 20-times longer pit LTs. In fibroblasts from individuals with CNM in which p.R465W dynamin is expressed at endogenous levels, we also observed the same effect of CCP flattening, but the LT of CPPs was only twice longer on average, supporting the hypothesis that abnormally high expression levels of dynamin may have detrimental effects. Flat p.R456W CCPs that we observed are similar to recently described FCLs (50) in their flat profile, prolonged LT, and stable association with dynamin. However, unlike FCLs that are heterogeneous in their shape, p.R465W CCPs retain their round shape through the entire LT. At the early stage of flattening, p.R465W CCPs also share geometrical similarity with clathrin-coated lattices, which were suggested as being capable of productive endocytosis (29). Significantly lower numbers of fully internalized CCPs in p.R465W skin fibroblasts found in TEM thin sections support our SICM-FCS observations that CME is disrupted in these cells.

Using correlative SICM-FCS, we found that p.R522H mutation also significantly increases the LT of CCPs when expressed in Cos-7 cells and in patient-derived skin fibroblasts. However, in contrast to p.R465W, p.R522H CCPs maintain their shape and close abruptly, indicating that productive endocytosis takes place. Thin-section TEM data also suggest that CME is not strongly affected by p.R522H mutation, showing the densities of internalized CCPs in p.R522H fibroblasts being slightly higher compared with control. This observation is counterintuitive considering significantly longer LT of CCPs and could only be explained by a concomitant defect in uncoating. In our SICM-FSC observations, we also found unusual clustering of CCPs. Such closely formed CCPs were reported in early studies of fibroblasts by quick-freeze EM (57). More recent studies on the dynamics of CCPs using total internal reflection fluorescence (TIRF) microscopy were mainly focused on characterization of individual, sparsely placed pits, possibly because of lack of resolving power. Clustering observed in our SICM-FCM experiments and EM images of metal replica from unroofed cells was not observed in Cos-7 cells expressing DNM2-R522H-GFP, suggesting that it is not the mutation *per se* that results in clustering of CCPs.

Our TEM images revealed high densities of membrane-bound and internalized caveoli in control and mutant human skin fibroblasts. The fact that we did not observe numerous indentations corresponding to caveolar openings in SICM topographical images could either be explained by tightly constricted caveolar necks, making the size of the opening below the resolution level of our SICM in its present setting, or the presence of stomatal diaphragms (Fig. 4*A*, right panel, asterisk) formed by PV1 protein encoded by plasmalemmal vesicle–associated protein gene in humans that is known to be expressed in skin (58). Such diaphragms that form at caveolar openings could physically prevent the SICM nanopipette from entering caveoli. Therefore, the spatial resolution of correlative SICM-FCS requires further improvement in order to make topographical imaging of caveoli possible.

In order to enable better comparison of our findings with previously published observations, we also measured the uptake of fluorescently labeled Tfn and CTxB and found that it is reduced in cells with p.R465W and p.R522H. Although uptake data support our findings at individual pit level and indicate that both CME levels are reduced by these mutations, the results have to be treated with caution because a substantial proportion of Tfn is uptaken by clathrin- and dynamin-independent mechanisms. Indeed, cells transfected with GTPase-deficient K44A mutant dynamin or treated with dynamin inhibitor Dynasore, often used as a negative control, on average show only 70% reduction in uptake at best (11, 22). This is compared with nearly complete absence of internalization on ice when all other remaining pathways are blocked (21). Such low specificity of fluorescently labeled cargo toward 1 particular uptake mechanism and the existence of alternative internalization pathways is known for currently used endocytic markers (*e.g.*, cholera toxin B) (59) and Tfn receptor (60, 61). Other nonspecific forms of uptake, such as micropinocytosis facilitated by membrane ruffles, in which dynamin is involved also contribute to the complexity. Indeed, it has previously been demonstrated that dynamin directly interacts with actin, and point mutations in the actin-binding domain cause aberrant membrane ruffling (62). In particular, it has been shown that p.R465W mutation significantly decreases the enrichment to the dorsal ruffle and supresses raft-dependent endocytosis; however, this does not affect the rate of macropinocytosis of horse radish peroxidase (23). It is also well documented that DNM2 plays a crucial role in macropinocytosis (63, 64) and acts as a transition controller for the recruitment of actin-related protein-2/3 (ARP2/3) complex activators required for IL-2 receptor endocytosis by membrane protrusion-assisted clathrin- and caveolin-independent mechanisms (65). Using SICM topographical imaging alone, we have demonstrated that mutations in DNM2 significantly reduce membrane ruffle density and therefore may affect nonselective uptake levels, further complicating the interpretation of uptake of fluorescent markers specific to particular types of endocytosis. This correlates well with the recently published observation that CNM-linked DNM2 mutations disrupt the formation of new actin filaments as well as the stimulus-induced translocation of glucose transporter 4 to the plasma membrane (66).

Our study suggests that CME and caveolae function are impaired by CNM-associated DNM2 mutations, which may have direct clinical relevance. On one hand, caveolae dysfunction was associated with a class of diseases called caveolinopathies, encompassing a wide range of clinical spectrum (67), including skeletal muscular diseases (68). In CNM, abnormal caveolin staining and accumulation of caveolae have already been reported in muscle biopsies from patients affected by the autosomal recessive form because mutations in the bridging integrator 1 (*BIN1*) gene encoding the Amphiphysin 2 (69, 70). We report here the first evidence that CNM-associated DNM2 mutations may also affect caveolae function. Further investigation will be necessary to confirm such a defect in muscle tissue and identify potential consequences on caveolae functions, including endocytosis, plasma membrane organization, signaling pathways, and mechanosensing. On the other hand, alteration of CME, a key regulator of turnover and function of membrane receptors and channels, may also lead to a deleterious impact on muscle function. A defect of clathrin-coated vesicles has been previously associated with several human diseases, including cancer, neurologic disorders, and myopathy (71, 72), and remains to be further investigated in centronuclear myopathies. In particular, CME defect may alter membrane content of ion channel, as suggested by alteration of the membrane permeability to calcium, leading to abnormal calcium homeostasis in a mouse model of the DNM2-linked CNM (73). A better knowledge of involvement of clathrin- and caveolin-dependent endocytosis in the pathomechanisms of the DNM2-linked CNM will be of particular interest for identification of specific targets for future development of therapeutic approaches.

Overall, in this study, we showed how SICM-FCM is able to establish a spatial and temporal correlation between the formation of the endocytic pit nanostructure and the recruitment of fluorescently tagged molecules responsible for the pit formation and scission. This is a highly important advance that makes dissecting the mechanistic action of membrane proteins possible in real time and at nanoscale. Here, we demonstrated the impairment of CME as potential pathomechanism in *DNM2*-related CNM at the structural level of individual CCPs in the most pertinent cell model (*i.e.*, patient-derived cells).

## Supplementary Material

This article includes supplemental data. Please visit *http://www.fasebj.org* to obtain this information.

## Abbreviations

3D: 3 dimensional
CCP: clathrin-coated pit
CLC: clathrin light chain
CNM: centronuclear myopathy
CME: clathrin-mediated endocytosis
CMT: Charcot-Marie-Tooth disease
CTxB: cholera enterotoxin subunit B
DNM2: dynamin 2
EM: electron microscopy
FCL: flat clathrin lattice
FCM: fluorescence confocal microscopy
FCS: fetal calf serum
GFP: green fluorescent protein
HSP: hereditary spastic paraplegia
LLSM: lattice light sheet microscopy
LT: lifetime
MD: middle domain
PH: pleckstrin-homology
TEM: transmission electron microscopy
SICM: scanning ion conductance microscopy
STED: stimulated emission depletion
Tfn: transferrin
WT: wild type
