# Supplementary material for: Correlative SICM-FCM reveals changes in morphology and kinetics of endocytic pits induced by disease-associated mutations in dynamin
Source: FASEB J. 2019 Apr 24;33(7):8504–18. doi: 10.1096/fj.201802635R (PMC6593877; doi:10.1096/fj.201802635R)
Supplement: Supplementary file 1 [file fj.201802635R.sd1.docx]

Supplemental Data


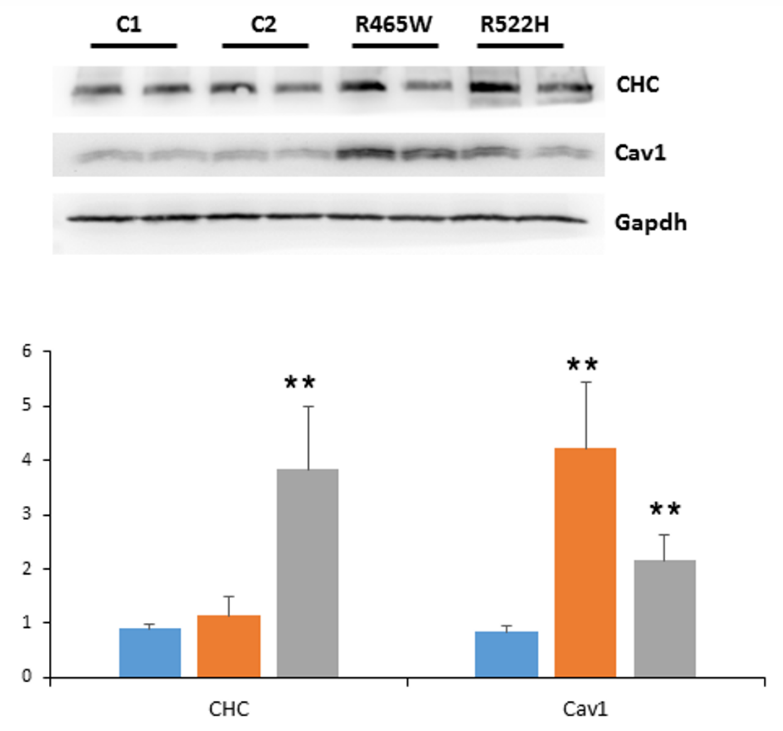


**Figure S1.** Quantitative comparative western blot analysis of clathrin and caveolin expression in human skin fibroblasts. Mean ± sem of 4 independent culture dishes. ** p<0.01 vs. control. Values for the 2 controls were pooled: n=8 for control and 4 for p.R465W and p.R522H.


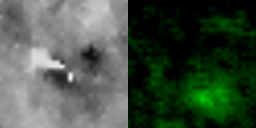


**Movie 1.** Nucleation, maturation and closure of CCP in Cos-7 cell transfected with DNM2-WT-GFP.


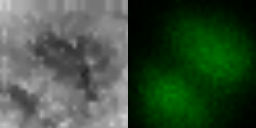


**Movie 2.** Nucleation, widening and disintegration of CCPs in Cos-7 cell transfected with mutant DNM2-R465W-GFP.


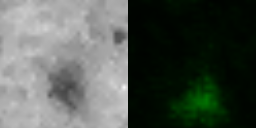


**Movie 3.** Nucleation, widening and disintegration of CCP in Cos-7 cell transfected with mutant DNM2-R522H-GFP.


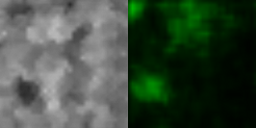


**Movie 4.** Nucleation, maturation and closure of CCP in skin fibroblast from healthy individual transfected with CLC-GFP.


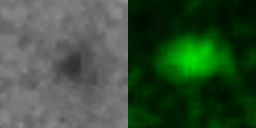


**Movie 5.** Nucleation, widening and disintegration of CCP in skin fibroblast from patient with p.R465W mutation transfected with CLC-GFP.


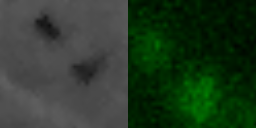


**Movie 6.** Nucleation, maturation and closure of CCPs forming cluster in skin fibroblast from patient with p.R522H mutation transfected with CLC-GFP.
